# The Role of Experimental Animal Models in Liver Transplantation Training Programs

**DOI:** 10.3390/biomedicines14030533

**Published:** 2026-02-27

**Authors:** Mohamed El-Shobari, Mahmoud M. Ramadan, Mohamed Tarek Mahdi, Ali Aljanaahi, Hasan A. Abo-Jouma, Ahmed Lamey

**Affiliations:** 1 Department of Clinical Sciences, College of Medicine, University of Sharjah, Sharjah 27272, United Arab Emirates; melshobari@sharjah.ac.ae (M.E.-S.); habojouma@sharjah.ac.ae (H.A.A.-J.); 2 Research Institute of Medical and Health Sciences, University of Sharjah, Sharjah 27272, United Arab Emirates; 3 Department of Cardiology, Faculty of Medicine, Mansoura University, Mansoura 35516, Egypt; 4 Department of Surgery, University Hospital of Sharjah, Sharjah 27272, United Arab Emirates; tmahdy@yahoo.com; 5 Graduate Medical Education Department, Mohammed Bin Rashid University of Medicine and Health Sciences, Dubai 505055, United Arab Emirates; amaljanaahi@dubaihealth.ae; 6 Department of General Surgery, Faculty of Medicine, Kafrelsheikh University, Kafrelsheikh 33516, Egypt; dr.ahmedlamey@gmail.com

**Keywords:** liver transplantation, surgical education, animal models, rodent, porcine, non-human primates, simulation, competency-based training

## Abstract

Liver transplantation (LTx) is among the most technically challenging procedures in abdominal surgery, especially for patients with severe liver fibrosis and cirrhosis. Constraints in surgical exposure, ethical considerations, and patient safety requirements have expedited the implementation of structured, competency-based training programs. Experimental animal models are crucial for advanced transplant training, offering physiological realism, procedural practice, and translational understanding of surgical problems associated with fibrosis. This narrative review critically synthesizes material from 1990 to 2024 sourced from PubMed/MEDLINE, Scopus, and Google Scholar, concentrating on the pedagogical utilization of rodent, porcine, and non-human primate models. We explicitly associate each model with specified training objectives, evaluative assessment instruments, and the incorporation of contemporary simulation technologies. The review additionally contrasts global training norms and suggests a unified, hybrid architecture to enhance skill acquisition, ethical governance, and patient outcomes.

## 1. Introduction

Liver transplantation (LTx) has transitioned from an experimental intervention to a conventional therapeutic approach for specific patients with end-stage liver disease, primarily resulting from advanced liver fibrosis and cirrhosis [1,2]. Fibrotic parenchyma, portal hypertension, and coagulopathy markedly elevate operative risk. Despite substantial advancements in surgical techniques, anesthesia, and immunosuppressive medication, LTx is associated with considerable perioperative risk, especially in the early postoperative phase [2,3]. The procedure requires expertise in intricate liver and blood vessel dissection, regulated blood flow management during the anhepatic stage, and careful rebuilding of blood vessels and bile ducts. Simultaneously, post-transplant success relies on the identification and control of immune-mediated damage, infections, and metabolic problems [1,3].

Training in LTx is progressively hindered by diminished surgical exposure, rigorous regulations, and the necessity to mitigate risks during the learning process [4,5]. Consequently, standardized training methodologies that provide safe repetition of critical stages have become indispensable. Experimental animal models have historically been fundamental to transplant training, allowing surgeons and interdisciplinary teams to refine technical abilities, enhance intraoperative decision-making, and improve coordination within a living physiological context.

This narrative review consolidates experimental and instructional material published mostly from 1990 to 2024, sourced from PubMed/MEDLINE, Scopus, and Google Scholar, concentrating on animal models specifically utilized for surgical training and competency enhancement in LTx. This paper analyzes the function of experimental animal models in LTx training programs, focusing on educational significance, constraints, ethical and regulatory considerations, and prospective incorporation with contemporary simulation technology.

## 2. Materials and Methods

### 2.1. Study Design

This manuscript is a narrative review aimed at synthesizing educational and experimental literature focused on animal models used specifically for training and competency development in LTx.

### 2.2. Literature Search Strategy

A comprehensive search of PubMed/MEDLINE, Scopus, and Google Scholar was performed to discover pertinent publications available until December 2024. Combine the search phrases “liver transplantation”, “animal models”, “surgical training”, “transplant education”, “rodent”, “porcine”, “non-human primate”, and “simulation.” Key papers’ reference lists were manually examined to identify other qualifying studies.

### 2.3. Eligibility Criteria

Studies were included if they focused on animal models used for training, education, procedural rehearsal, or competency development in the context of LTx. Purely mechanistic or laboratory studies without educational relevance were excluded unless they provided directly transferable methodological insights.

### 2.4. Classification of Experimental Models

Models were categorized by species and by training application. Broadly, rodent models are most often used for microsurgical skill development and immunobiology, porcine models for high-fidelity open and minimally invasive procedural simulation, and non-human primates for advanced translational immunology and specialized training contexts [3,6,7].

### 2.5. Ethical Considerations

This study, which examines already available material, did not necessitate institutional ethical approval or informed consent.

## 3. Importance of Training in Liver Transplant Programs

LTx necessitates a proficient multidisciplinary team and expertise in surgical techniques, perioperative critical care, and transplant immunology [1,5]. Early post-transplant mortality is predominantly influenced by sepsis, graft malfunction, and perioperative complications, highlighting the necessity for systematic training and team preparedness [2,5]. Experimental training platforms provide the systematic, supervised practice of procurement, implantation, and reconstructive procedures prior to clinical implementation, thus minimizing technical errors and enhancing procedural efficiency during the learning phase [4].

## 4. Educational Role of Experimental Animal Models

The global demand for LTx continues to increase globally, while access to high-volume transplant centers and case-based learning opportunities remains uneven. Animal models provide a structured environment for iterative practice, objective feedback, and validation of new operative strategies, including preservation approaches and perioperative protocols [4,5]. They also facilitate non-technical training, communication, leadership, and crisis resource management, particularly when used in integrated team simulations [5]. The alignment between experimental species and defined educational objectives in liver transplant training is summarized in Table 1.

## 5. Categories of Experimental Animal Models Used in Liver Transplantation Training

Although numerous animal models stem from experimental research, their instructional use fundamentally diverges in objectives, evaluation criteria, and ethical rationale. The emphasis on training-oriented applications prioritizes reproducibility, skill development, and team efficacy over hypothesis-driven biological exploration.

### 5.1. Rodent Models

Rodent models are extensively utilized in transplantation laboratories because to their cost-effectiveness, ease of maintenance, and appropriateness for repetitive procedures and high-throughput research. The rat model is a recognized platform for orthotopic LTx among rodents, extensively utilized to enhance microsurgical techniques and examine the immunological milieu of liver allografts [3]. Rodent transplantation models facilitate the incremental development of competencies such as vascular manipulation, micro-anastomosis, and gentle tissue handling, which are applicable to clinical transplant surgery. Moreover, rodent allotransplantation provides a regulated framework for investigating rejection biology and immunomodulatory approaches, which is impractical due to the restricted availability of human tissue [3,8].

### 5.2. Porcine Models

Porcine models are often considered the optimal platform for high-fidelity transplant surgery training due to their anatomical and physiological resemblance to humans and similar organ size. These attributes provide an authentic simulation of donor hepatectomy, organ procurement, back-table preparation, vascular repair, and biliary anastomosis. Pigs are compatible with conventional clinical imaging technologies, facilitating procedure planning and postoperative evaluation during training [9]. Practical benefits encompass widespread accessibility and reasonable acquisition and maintenance expenses compared to non-human primates [7,9].

### 5.3. Non-Human Primate Models

Non-human primates (NHPs) offer the greatest translational significance for immunology and long-term graft outcomes due of their strong phylogenetic relationship with humans [6]. NHP transplantation models have provided significant insights into allograft immune responses and the assessment of immunosuppressive treatments. Studies in rhesus models have identified cellular immune profiles linked to rejection, thereby enhancing translational immunological monitoring and mechanistic comprehension pertinent to clinical practice [10]. NHP models are generally confined to advanced research and specific, specialized training environments due to significant ethical, regulatory, and financial limitations [6]. A comparative overview of species characteristics, fibrosis induction strategies, and translational relevance is summarized in Table 2.

## 6. Advantages of Animal Models in Training

### 6.1. Pragmatic Surgical Scenarios

Animal models provide comprehensive procedural rehearsal encompassing procurement, implantation, and reconstruction phases. This facilitates intentional rehearsal of time-sensitive maneuvers, organized team processes, and the handling of unforeseen intraoperative occurrences. Systematic studies of simulation in transplant surgery indicate that hands-on models enhance technical performance and confidence, especially when integrated into structured curriculum [4].

### 6.2. Physiological Relevance

In contrast to synthetic or static models, live animal platforms offer genuine physiological responses, encompassing pulsatile flow, coagulopathy, hemodynamic instability, and metabolic reactions. This realism allows trainees to practice hemostasis, vascular control, ischemia management, and anesthetic coordination in situations that closely resemble clinical transplantation. A structured comparison of model fidelity, cost, and recommended trainee level is presented in Table 3.

## 7. Limitations (Constraints) of Animal Models in Training

### 7.1. Species Differences

Species-specific anatomy, coagulation physiology, and immunological responses restrict straightforward extrapolation from animal training models to human transplantation. Rodent models facilitate microsurgical replication but significantly differ from human-scale dissection and repair. In contrast, large-animal models more accurately reflect human anatomy, although they may exhibit variations in biliary and vascular structures as well as differences in immune-mediated damage responses [6,7].

### 7.2. Resource, Ethics, and Access Constraints (Translational Gap)

Animal-based training necessitates infrastructure, specialized people, regulatory adherence, ethical considerations, and substantial ongoing expenses, thus restricting access to continuous curricula. These limits underscore the necessity for objective training requirements evaluations, meticulous model selection aligned with learning objectives, and the integration of alternatives that can minimize animal usage [4,11].

## 8. Ethical and Regulatory Considerations in Animal Research

The ethical governance of animal utilization in biomedical research has significantly improved in response to social expectations and advancements in animal welfare science. Various jurisdictions prioritize the implementation of the 3Rs (replacement, reduction, and refinement) in conjunction with anticipated project evaluations and human competency standards [11]. In transplant training, ethical acceptability relies on explicit educational justification, reduction in pain, compliance with standardized anesthetic and humane endpoints, and adequate oversight. Where possible, curricula have to integrate alternatives such ex vivo systems and proven simulations to minimize animal usage while maintaining training efficacy [4,11].

## 9. Evidence from Training Initiatives and Outcomes

Published publications delineate various transplant training curricula that utilize animal models, frequently integrating theoretical instruction with practical laboratory components. A systematic assessment of technical skills simulation in transplant surgery indicated that structured courses utilizing biological and simulation-based platforms enhanced trainee performance measures and self-reported confidence [4]. Historical program data indicate that supervised training in clinical LTx does not inherently jeopardize patient outcomes, provided that systematic oversight and competency advancement are upheld [12,13,14].

## 10. Evaluation of Training Outcomes

### 10.1. Skills Acquisition and Competency

Competency-based transplant education necessitates the objective evaluation of procedural milestones (e.g., quality of vascular anastomosis, control of ischemia time, recognition of errors) and non-technical abilities (communication, leadership, situational awareness). Validated evaluation instruments exhibit variability between programs, necessitating the wider implementation of standardized metrics to facilitate benchmarking and curricular enhancement [4,5].

### 10.2. Program Efficacy and Patient Outcomes

Transplant training programs should be assessed not only for individual skill acquisition but also within the wider contexts of quality, encompassing institutional structures, outcome monitoring, and ongoing enhancement. Evaluations of quality domains among transplant programs highlight discrepancies in performance among centers and emphasize the necessity of consistent assessment systems [15].

### 10.3. Objective Validation and Assessment Tools in Liver Transplant Training

Liver transplant training programs are increasingly utilizing objective, validated assessment measures to enhance educational efficacy, moving away from reliance on subjective self-reporting alone. Commonly utilized metrics encompass procedure-specific checklists, worldwide rating scales like the Objective Structured Assessment of Technical Skills (OSATS), time-to-completion standards, error rates, and the quality of anastomoses evaluated through patency testing or leak pressure measures [15].

In animal-based training, physiological endpoints such as hemodynamic stability, ischemia duration, bile output, and graft morphology serve as objective indications of procedural proficiency. These measures improve reliability and repeatability when integrated with video-based review and blinded expert evaluation.

The integration of these tools into competency-based curricula facilitates a systematic advancement from simulation to animal models and, finally, to supervised clinical practice, hence enhancing transparent credentialing and quality assurance across training programs. Competency domains and their associated objective assessment metrics are summarized in Table 4.

## 11. Future Directions in Training Programs

### 11.1. Technological Progressions

Advances in virtual reality, augmented reality, and 3D printing have expanded opportunities for scalable, repeatable skills training outside the operating room.

### 11.2. Incorporation of Animal Models and Sophisticated Simulation Technologies

**Figure 1:**
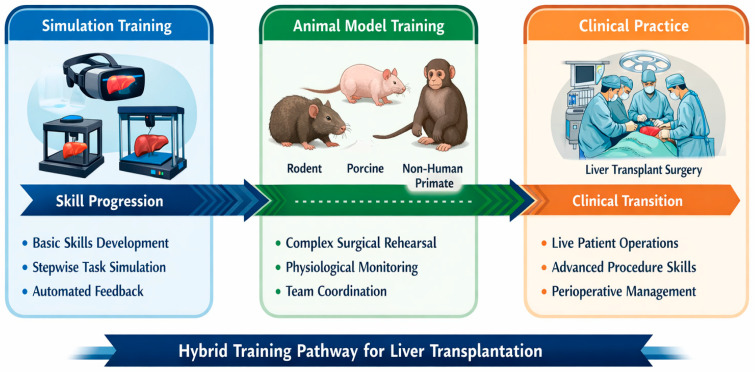
Integrated Hybrid Training Pathway. Early-phase training using virtual reality and 3D-printed simulators → Intermediate ex vivo and synthetic models → Advanced animal model-based physiological training → Supervised clinical LTx.

Animal models and simulation-based technology should be incorporated into a cohesive educational continuum rather than operating as separate modalities. Virtual reality and 3D-printed models are optimally designed for initial training phases, facilitating repeated practice of anatomical orientation, suturing, and procedural sequencing without ethical or logistical limitations (Figure 1) [4,5].

Animal models thereafter offer the physiological realism essential for further training stages, encompassing the treatment of hemorrhage, ischemia–reperfusion injury, and anesthetic coordination. This orchestrated integration enhances educational outcomes while complying with ethical norms of reduction and refinement [11] (Figure 1).

## 12. Standardization of International Training and Global Harmonization

Standards for liver transplant training differ significantly among locales. In Europe, training programs are often organized by national transplant organizations and societies affiliated with the European Liver and Intestine Transplant Association (ELITA), focusing on structured fellowships and centralized accreditation. In the United States, schools are governed by the Accreditation Council for Graduate Medical Education (ACGME), which prioritizes case-volume criteria and milestone-oriented evaluation.

Conversely, several Asian nations utilize hybrid apprenticeship-simulation models, frequently limited by inconsistent availability to high-capacity transplant centers. These variations highlight the necessity for globally standardized benchmarks that delineate essential competencies rather than solely relying on case numbers.

Experimental animal models, when integrated with standardized evaluation tools, can function as an impartial platform for global benchmarking, facilitating objective comparisons of skill gain regardless of regional resource disparities [10].

## 13. Conclusions

Experimental animal models are essential for LTx training as they offer high-fidelity procedural practice and physiological realism that non-biological simulations cannot yet fully imitate. Rodent models facilitate the enhancement of microsurgical skills and immunology training, porcine models provide human-scale surgical simulation, and non-human primates yield specific insights into translational immunology. Nonetheless, ethical, regulatory, and budgetary limitations need careful model selection and greater use of established simulation alternatives. Future training programs will likely be refined through hybrid, competency-based approaches that integrate contemporary simulation technologies with the strategic application of animal models to improve surgeon readiness and safeguard patient outcomes.

## Figures and Tables

**Table 1:** Alignment of Animal Models with Liver Transplant Training Objectives.

Model	Primary Training Objectives	Strengths	Limitations
Rodent	Microsurgical skills, immunology	Low cost, high repetition	Scale differences
Porcine	Full procedural simulation	Human-scale anatomy	Resource intensive
Non-human primate	Translational immunology	Closest immune similarity	Ethical and cost constraints

**Table 2:** Comparative analysis of experimental animal species used in fibrosis-oriented transplant training.

Species	Fibrosis Induction	Training Application	Advantages	Limitations	Translational Value
Rat	CCl_4_, bile duct ligation	Microsurgery, immunology	Low cost, reproducibility	Small size	Mechanistic
Pig	Surgical + chemical	Full transplant simulation	Anatomical realism	Cost, ethics	High
Primate	Immune-mediated	Chronic rejection	Immune similarity	Ethical constraints	Very high

**Table 3:** Comparison of experimental models for LTx training.

Feature	Rodent	Porcine	Non-Human Primate
Anatomical fidelity	Low–moderate	High	Very high
Physiological realism	Low	High	Very high
Microsurgical skill training	Excellent	Limited	Limited
Hemodynamic management training	Poor	Excellent	Excellent
Team-based OR training	Poor	Excellent	Excellent
Immunological similarity to humans	Moderate	Moderate	High
Cost	Low	High	Very high
Ethical/regulatory burden	Low–moderate	High	Very high
Availability	High	Moderate	Limited
Recommended trainee level	Early	Intermediate–advanced	Advanced/research

**Table 4:** Competency domains and assessment metrics in experimental LTx training.

Domain	Skills	Assessment Tools
Technical	Anastomosis quality, bleeding control, warm ischemia time	OSATS, time-to-completion, leak testing, error scoring
Physiological	Hemodynamic stability, transfusion requirement, metabolic control	MAP variability, lactate trends, survival time
Non-technical	Leadership, communication, situational awareness	NOTSS, teamwork rating scales, crisis simulations

## Data Availability

The original contributions presented in this study are included in the article. Further inquiries can be directed to the corresponding author.
